# How the Protein Data Bank changed biology: An introduction to the JBC Reviews thematic series, part 2

**DOI:** 10.1016/j.jbc.2021.100748

**Published:** 2021-05-03

**Authors:** Lila M. Gierasch, Helen M. Berman

**Affiliations:** 1 Departments of Biochemistry & Molecular Biology and Chemistry, University of Massachusetts, Amherst, Amherst, Massachusetts, USA; 2 Department of Chemistry and Chemical Biology, Rutgers, The State University of New Jersey, Piscataway, New Jersey, USA; 3 Department of Biological Sciences and Bridge Institute, University of Southern California, Los Angeles, California, USA

**Keywords:** crystal structure, cryo-electron microscopy, protein structure, nucleic acid, nucleic acid structure, structural genomics, NMR, integrative modeling

## Abstract

In part 1 of this remarkable collection, we told you the story of The Protein Data Bank (PDB) (1), which was founded 50 years ago, and we illustrated the breadth of the science contained within it with ten informative review articles. The second half of this collection is a continuation of our celebrations to mark this momentous anniversary. Part 2 provides eight more superb articles describing how the PDB has influenced biology over the course of the last half-century and how biology has fueled the deposition of impactful structures in the PDB. Here are some brief synopses of the articles you will enjoy in part 2!

Our understanding of cellular signaling is dependent on the knowledge of key protein structures. In their review, **Susan S. Taylor** (University of California, San Diego) and colleagues use cAMP-dependent protein kinase as a model system to show us the many levels of detail of the structural basis of signaling (2). The authors describe the landmark structure of the catalytic subunit of PKA and then explore how the kinase domain is packaged in an inactive state by the cAMP binding regulatory subunits. The review also captures the importance of understanding crystal packing, using multiple structural determination methods, and thinking outside the box.

Transcriptional regulation is fundamental to cellular homeostasis, coordinating responses to a wide array of physiological signals. In her review, **Cynthia Wolberger** (The Johns Hopkins University School of Medicine) focuses on how the structures of protein–DNA complexes have provided insights into regulation of transcription (3). She describes some of the earliest structures that were determined in the 1980s and explores the structural basis for the remarkable diversity of sequence-specific binding modes. She then discusses the multimeric complexes that are utilized in eukaryotic systems and the ability of cryo-electron microscopy to enable visualization of very large macromolecular complexes with ever-increasing molecular detail, now facilitating the structural understanding of large transcription and elongation machines.

The field of structural immunology was born 50 years ago with the first determination of antibody structures. In their review, **Ian Wilson** (The Scripps Research Institute) and his colleague Robyn Stanfield trace how structures were pivotal to our understanding of antibody–antigen interactions and revealed the diversity of the antigen receptors and the antigens in the immune system (4). They describe the wealth of structural information we have gathered about viral antigens of enveloped viruses, which are responsible for life-threatening diseases such as influenza, HIV, and COVID-19. The way in which the experimental challenges of sample preparation and structure determination have been overcome highlights the progress that has enabled structural biology to be such an effective method for understanding health and disease.

Another field of biological science that has emerged hand in hand with the PDB is protein homeostasis—the array of cellular networks dedicated to maintaining the health of the proteome. **Helen Saibil** (Birkbeck College) provides an exciting and informative review of the evolution of the field of protein homeostasis, from the first structures of chaperonins to the recent elucidation of protein aggregate structures (5). In parallel, she describes how the determination of these structures has relied increasingly on cryo-electron microscopy. Recent exciting advances that are opening doors to the complex cellular physiology of protein homeostasis include complexes containing multiple chaperones along with their substrates and emerging methods to observe protein homeostasis machines in their cellular context.

Structural Genomics (SG) as a field unto itself was an outgrowth of the huge amount of data produced by the genome sequencing projects and the resultant challenge of determining the structures of the proteins encoded by every genome. **Andrzej Joachimiak** (Argonne National Laboratory and University of Chicago) and his colleague Karolina Michalska describe how worldwide structural genomics programs attempted to meet this challenge, developing high-throughput pipelines for all steps en route to structural characterization (6). These efforts have resulted in a large number of unique structures and vastly improved methods for all of structural biology.

Traditionally, single methods including X-ray crystallography, NMR spectroscopy, and cryo-electron microscopy were deployed to elucidate macromolecular structures. In recent years, it has been recognized that the data from multiple experimental methods combined with computational modeling empower the determination of integrative models of large macromolecular machines. **Andrej Sali** (Research Collaboratory for Structural Bioinformatics Protein Data Bank and University of California San Francisco) describes how this type of integrative modeling using results from multiple approaches is done, the challenges it presents, and how a pipeline is being created for archiving these data (7). He ends his review by describing the future of integrative “metamodeling,” showing how it can be used to model an entire cell.

An inadvertent and undesirable outcome from presenting the beautiful structures in the PDB is that they can create an impression that macromolecules are static. **George Phillips** (Rice University) and his colleague Mitchell Miller dispel this notion, examining the ways in which biomolecular dynamics are studied (8). As case studies, they explore the numerous methods that have been used to analyze the binding of gaseous ligands to myoglobin and explore its overall dynamics, as well as the many different analyses of adenylate kinase that have led to a better understanding of its mechanism of catalysis. The use of both free electron lasers and cryo-electron microscopy has made it possible to get much deeper insight into dynamics. A new challenge will be how to best archive protein dynamics data so they will be useful for further research.

The last review in our collection chronicles how the visualization of macromolecular structure depiction evolved hand in hand with the PDB and advanced the ability of researchers to appreciate and functionally interpret molecular architecture. **Jane and David Richardson** (Duke University) and **David Goodsell** (The Scripps Research Institute and Research Collaboratory for Structural Bioinformatics Protein Data Bank) have together changed how we think of macromolecular structure and how we use images to better understand the biological processes we seek to elucidate (9). What is even more fun and informative, particularly to those who were not privy to the developments in structure visualization as they emerged, is to see the artistry that underlies the human appreciation for complex three-dimensional molecular structures.

The compilation of wonderful reviews in this second part of our thematic JBC issue celebrating the 50th anniversary of the PDB, together with the reviews included in part 1, illustrates the spectrum of hugely impressive science enabled by the establishment of the PDB to facilitate open sharing of macromolecular structures. Moreover, it is abundantly clear that the advances in biological science in which the PDB played a role also led to a synergistic evolution of the PDB to serve the scientific community in ever better ways. There is so much to celebrate! And so much wonderful science to embrace! We hope you enjoy the terrific articles in this collection as much as we do.

## Conflict of interest

The authors declare that they have no conflicts of interest with the contents of this article.
